# Source Apportionment of Polycyclic Aromatic Hydrocarbons in Sediment by the Application of Non-Negative Factor Analysis: A Case Study of Dalian Bay

**DOI:** 10.3390/ijerph15040761

**Published:** 2018-04-16

**Authors:** Fu-Lin Tian, Fa-Yun Li, De-Gao Wang, Yan-Jie Wang

**Affiliations:** 1 Institute of Eco-Environmental Sciences, Liaoning Shihua University, Fushun 113001, China; fltian@sina.com (F.-L.T.); yanjie_wang0810@hotmail.com (Y.-J.W.); 2 National & Local United Engineering Laboratory of Petroleum Chemical Process Operation, Optimization and Energy Conservation Technology, Liaoning Shihua University, Fushun 113001, China; 3 School of Environmental Science and Engineering, Dalian Maritime University, Dalian 116026, China; degaowang@163.com

**Keywords:** receptor model, statistical analysis, coastal area, organic pollutants

## Abstract

An improved method, factor analysis with non-negative constraints (FA-NNC) was adopted to apportion the sources of sediment polycyclic aromatic hydrocarbons (PAHs) in Dalian Bay, China. Cosine similarity and Monte Carlo uncertainty analysis were used to assist the FA-NNC source resolution. The results identified three sources for PAHs, which were overall traffic, diesel engine emissions and residential coal combustion. The contributions of these sources were quantified as 78 ± 4.6% from overall traffic, 12 ± 3.2% from diesel engine emissions, and 10 ± 1.9% from residential coal combustion. The results from the Monte Carlo uncertainty analysis indicated that the model was robust and convergent.

## 1. Introduction

Polycyclic aromatic hydrocarbons (PAHs), with two or more fused aromatic rings, are ubiquitous semi-volatile organic pollutants which are a significant public health and environmental concern [1]. PAHs mainly originate from the incomplete combustion of organic matters, such as the combustion of fossil fuels in vehicular engines, power generation from fossil fuels, biomass burning, incineration of industrial and domestic wastes, oil refinery and chemical engineering processes [2]. PAHs associated with air particulates, emitted from emission sources, are transported in the atmosphere and deposited through dry and wet deposition [3]. Once deposited, PAHs tend to sorb into soil or sediment for a long period of time, particularly for high molecular weight PAHs. Considering their degradation and unique source profiles, PAHs can serve as tracers of pollution sources [4]. 

Receptor models have been used to apportion the contributions of PAHs from emission sources based on observations at sampling sites [5,6,7,8]. An advanced receptor model, factor analysis model with non-negative constraints (FA-NNC), has been adopted for the source apportionment of PAHs in various environmental media [9,10,11,12,13]. The application of the FA-NNC model mainly depends on a large amount of sampling and precisely and accurately experimental analysis. The sediment PAH concentrations, which are used as the input parameters of the model, are inherently variable [14]. Environmental variability and error result in the deviation of PAH concentrations. Thus, to assess the efficiency and accuracy of the model prediction, uncertainty analysis should be adopted to estimate the reliability of model outputs [15,16]. However, few studies have combined receptor models with Monte Carlo uncertainty analysis successfully. In some cases [4,9,10], a few convergent datasets, rather than a large number of random normal distribution input parameters, were considered. In other cases [13,17,18], Monte Carlo simulation was used for model validation instead of uncertainty analysis.

The objective of this study is to determine the major PAH sources and contributions to the sediments of Dalian Bay, using the FA-NNC model combined with Monte Carlo uncertainty analysis. Cosine similarity was adopted to compare the differences between modeled and literature PAH source profiles [4,19]. To our knowledge, this is the first application of the FA-NNC model combined with Monte Carlo uncertainty and cosine similarity analysis. 

## 2. Materials and Methods

### 2.1. Study Area and Data

**Figure 1:**
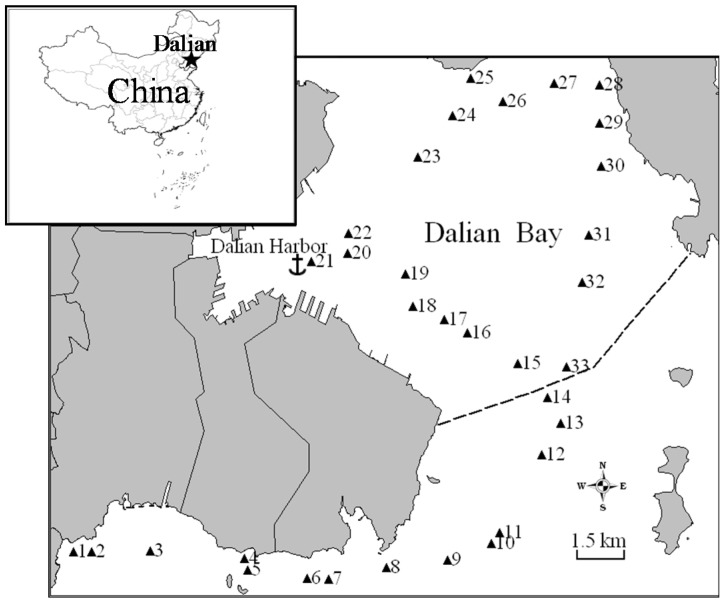
The sampling locations around the Dalian coastal areas. The dashed line represents the border of Dalian Bay.

Dalian is a typical coastal city situated in the northeastern monsoonal area of China (120°58′–123°31′ E, 38°43′–40°10′ N). Dalian Bay is located on the southeast side of the Liaodong Peninsula (Figure 1). The bay is open to the Yellow Sea in the east. Port of Dalian lies along the southern shore of the bay. Dalian Bay is ice-free all year-round, so it is one of the most convenient and busiest seaports of China. In our previous work [20,21], 33 surface sediment samples were collected concurrently at Dalian Bay in October 2008, and the concentrations of 15 priority PAHs (Table 1) in the sediment were analyzed. The locations of the sampling sites are shown in Figure 1. The sample from sampling site 21 is excluded from the data analysis because the PAH concentrations of this sampling site are extremely high, which may be impacted by evident point sources. Thus, the dataset consisting of 32 sediment samples and 15 PAHs were adopted in this study.

### 2.2. FA-NNC Model

The fundamental equation underlying the FA-NNC model is

(1) $$D_{m\times r}=C_{m\times n}\cdot R_{n\times r}$$

where the concentration matrix ***D*** is fractioned into the factor loading matrix ***C*** and the factor score matrix ***R***. The ***C*** matrix represents source profiles, and ***R*** represents source contributions. *m*, *n*, and *r* stand for the number of compounds, sources, and samples, respectively.

The FA-NNC model described by Rachdawong and Christensen [17] were followed. In briefly, data matrix ***D*** was averagely scaled to reduce the effects of bias from compounds with high concentrations. Factor loading matrix ***C*** and factor score matrix ***R*** were determined based on the computation method described by Imamoglu and Christensen [22]. The reduced ***C*** and ***R*** matrices were rotated with nonnegative constraints until the sum of square of the negative entries in the back-scaled ***C*** matrix was below 0.0001, which indicated that the iteration process is complete [23]. The coefficient of determination (COD), cumulative percent variance, Exner function, and convergence of the nonnegative rotations were all considered for determination of the significant factors. Naphthalene was not included in the modeling because of the high uncertainties during the entire experimental analysis and in its source fingerprints [19].

### 2.3. Cosine Similarity

Cosine similarity is a measure of similarity between two non-zero vectors [24]. Two vectors with the same orientation have a cosine similarity of 1; two vectors at 90° have a similarity of 0. The result of cosine similarity is neatly bounded between 0 and 1. The governing equation is

(2) $$\mathrm{Cosine}\;\mathrm{similarity}=\frac{A\cdot B}{\left\|A\|\cdot \|B\right\|}=\frac{\sum_{i=1}^{n}A_{i}B_{i}}{\sqrt{\sum_{i=1}^{n}A_{i}^{2}}\sqrt{\sum_{i=1}^{n}B_{i}^{2}}}$$

where ***A*** and ***B*** are modeled and literature PAH profiles, respectively.

### 2.4. Monte Carlo Uncertainty Analysis

Monte Carlo uncertainty analysis was adopted to calculate disturbance of the inputs through the model. As reported by Tao et al. [14] and Cao et al. [15], a normal distribution was used for all input parameters under consideration. The simulation repeated 1000 times, with new values randomly selected for all PAH compounds and samples [25].

The governing equation is as follows,

(3) $$D_{ij}=A_{ij}+C_{ij}A_{ij}\left[\sqrt{2}erf^{-1}\left(2R_{ij}-1\right)\right]$$

where ***D****_ij_* is the generated PAH concentration from PAH *i* and sample *j*, ***A****_ij_* is the measured concentration of PAH *i* from sample *j*, ***C****_ij_* is the coefficient of variation of PAH *i* from sample *j*, erf^−1^ is the inverse Gaussian error function, and ***R****_ij_* is a random number between 0 and 1 with a normal distribution.

## 3. Results and Discussion

### 3.1. Diagnostic Tools Application 

The purpose of FA-NNC is to represent the total variability of the original PAH data in a minimum number of factors. The coefficient of determination (COD), cumulative percent variance, and Exner function are diagnostic tools which can be used to determine the number of factors. Table 1 shows the results of diagnostic tools application for the sediment of Dalian Bay. Each element in Table 1 is determined by running the FA model without non-negative rotations, but with average scaling and back-scaling. It is generally considered to be an excellent correlation [26,27], while the Exner function value is lower than 0.1. Diagnostic tool results show that the Exner function is below 0.10, and the cumulative percent variance is greater than 95% for all but one factor solution, which represents excellent correlation.

COD values for the two-factor solution are greater than 0.8 except for AcNP (acenaphthylene) and BghiP (benzo[g,h,i]perylene). The COD values for AcNP, FlA (fluoranthene), IP (indeno[1,2,3-cd]pyrene) and BghiP become more determined by adding a third factor. Addition of the fourth factor improved the coefficient of determination and cumulative variance marginally. The diagnostic tools indicated that two or three factors are reasonable in this case. 

### 3.2. Uncertainty Analysis

In the previous studies, Monte Carlo uncertainty analysis was used to evaluate the overall uncertainty in the field of multi-media fate modeling [14,15]. In addition, it was also used to evaluate the uncertainties of factor analysis with non-negative constraints with restricted artificial dataset [9]. However, few studies have successfully applied Monte Carlo analysis to real world dataset in the field of PAH source identification. In order to systematically evaluate the uncertainties of receptor modeling, a programming loop can be used. In this research, a computer programming was applied to simulate the uncertainties derived from a large number of random normal distribution input parameters, rather than a few artificial data matrices.

**Figure 2:**
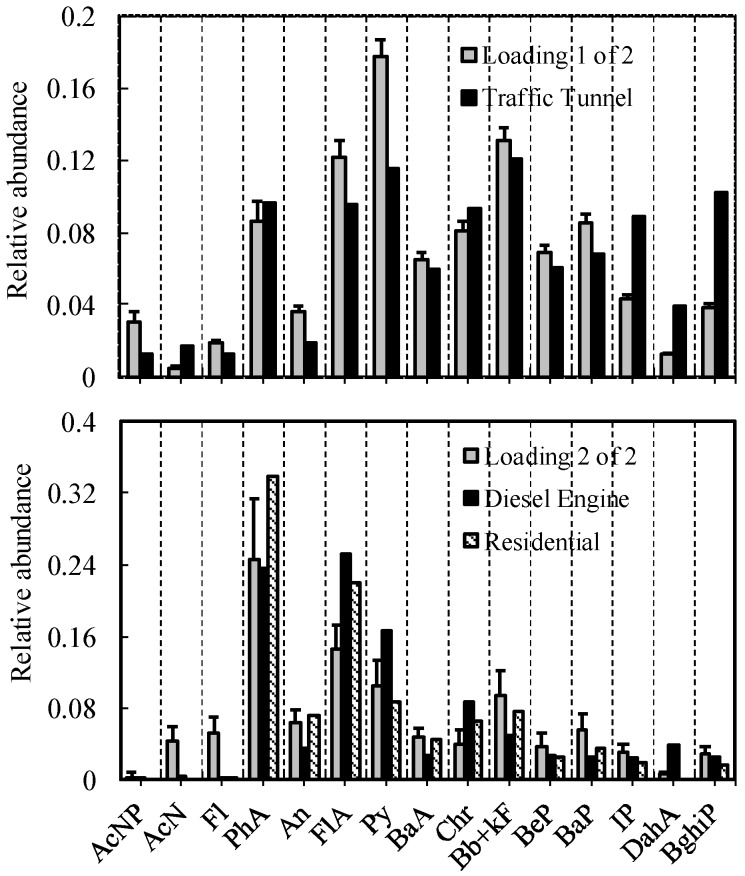
Factor loading plots of sediment PAH two-source factor analysis solutions with error bars representing the standard deviation of the mean derived from the Monte Carlo uncertainty analysis.

**Figure 3:**
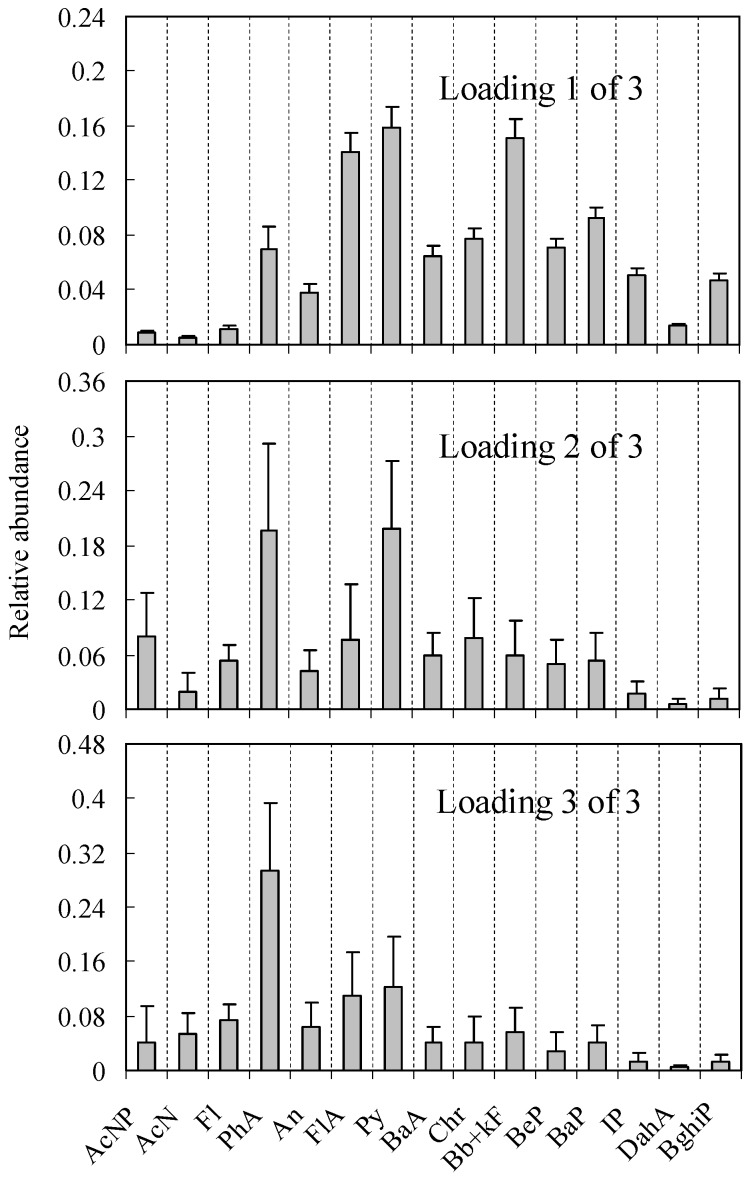
Factor loading plots of sediment PAH three-source factor analysis solutions with error bars representing the standard deviation of the mean derived from the Monte Carlo uncertainty analysis.

The sediment PAH concentrations are inherently variable. Thus, it is necessary to evaluate the overall uncertainty and variability in receptor modeling. In this research, a computer program was used to model the uncertainties derived from 1000 times program loops, rather than a few artificial data matrices. The standard deviation of the resulting factor loadings and factor scores were calculated to be the uncertainties, which are shown in Figure 2 and Figure 3.

### 3.3. FA-NNC Model Performance

The two-source factor loading solution presented in Figure 2 displays two PAH source profiles. The first factor that accounts for 90.70% of the total variance was predominantly loaded on PhA (phenanthrene), FlA, Py (pyrene), Bb + kF (benzo[b] + [k]fluoranthene), BaP (benzo[a]pyrene) and BghiP. By visually comparing PAH patterns and from the cosine similarity between modeled and literature PAH profiles [4,19], the first factor appears to be from traffic-related sources. This loading pattern is also similar to the loading 2 of 2, which was defined as being from a traffic tunnel source, as reported by Bzdusek et al. (2004). The second factor that accounts for 5.12% of the total variance was predominantly loaded on PhA, FlA and Py. This appears to be a mix of residential coal combustion and diesel engine profiles. The percent contribution from traffic-related sources and residential coal/diesel engine was calculated to be 80% and 20% (±8.6%), respectively, based on the average of factor scores by the uncertainty analysis.

By adding a third factor, loading 2 of 2 was separated into two factor loadings, loading 2 of 3 and loading 3 of 3. Loading 2 of 3 has a higher Py composition than loading 3 of 3. Source profiles of the three-factor solution for the sediment of Dalian Bay are displayed in Figure 3. By comparing between the literature source profiles and the cosine similarity shown in Table 2, the three sources include a traffic tunnel source, a diesel engine source and a residential coal source. Loading 1 of 3 is a traffic tunnel profile. As shown in Figure 3, the model reproduces the loading profile very well. This is also supported by the cosine similarity calculations (Table 2) for a traffic tunnel and with the low model uncertainties. 

Loading 2 of 3 appears to be a diesel engine profile. The FA-NNC model does not reconstruct AcNP, FlA and Py to the fractional source compositions as described in the literature. Monte Carlo uncertainty analysis indicates high uncertainties of AcNP, FlA and Py compositions in the loading profiles. 

Loading 3 of 3 has properties of a residential coal profile. The model reproduces the profile accurately, as supported by the cosine similarity calculation (Table 2) for residential coal, which is the highest. Loading 3 of 3 and loading 2 of 3 have similar loading patterns. These two loading profiles show considerable similarities, which resulted in high uncertainties in the modeling outputs.

**Figure 4:**
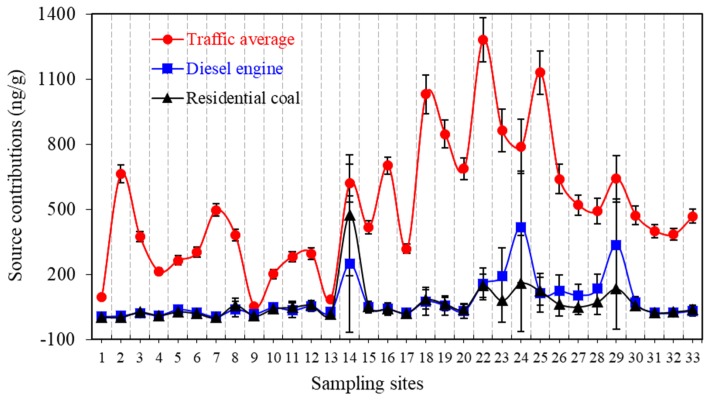
Contributions of each factor to total sediment PAH concentrations at each sampling site in Dalian Bay with error bars representing the standard deviation of the mean derived from the Monte Carlo uncertainty analysis.

Source contributions for the two-source solution are consistent with the three-source solution. Thus, only the three-source contributions are shown in Figure 4 and discussed below. Source contributions of the three-factor solutions indicate contributions from the different PAH sources to the total PAH concentrations over the 32 sampling sites. The highest factor scores are observed at the Dalian Harbor area (sampling site 22) for all factor scores. As shown in Figure 4, traffic average source contributions, varying from 46% to 99%, are dominant in all the sampling sites, particularly in the harbor area (sampling sites 16–25). Additionally, uncertainty analysis indicates that the standard deviations are relatively low and more stable for the source contributions of the traffic average. For diesel engine and residential coal, the standard deviations of the source contributions are extremely high and fluctuate greatly, especially at sampling sites 14, 23, 24 and 29. At these sampling sites, the source contributions of diesel engines and residential coal are relatively high. As discussed previously, the two loading profiles show considerable similarities, which resulted in high uncertainties of the source contributions. Overall, the main sources of PAHs were overall traffic (78 ± 4.6%), diesel engine emissions (12 ± 3.2%) and residential coal combustion (10 ± 1.9%). 

## 4. Conclusions

The FA-NNC model combined with Monte Carlo uncertainty analysis and cosine similarity calculations was adopted successfully to elucidate the source apportionment of PAHs in sediment of Dalian Bay. Regional variations clearly impacted the sources of PAHs in sediment. According to the cosine similarity calculations between the modeled and literature source profiles, the main sources for PAHs are as follows: overall traffic (78 ± 4.6%), diesel engine emissions (12 ± 3.2%) and residential coal combustion (10 ± 1.9%). The two-source solution, traffic-related sources (80%) and residential coal/diesel engine (20%), are consistent with the three-source solution. Monte Carlo uncertainty analysis indicated that similar source profiles would result in high uncertainties for modeling outputs.

## Figures and Tables

**Table 1:** Results of diagnostic tools application for the determination of the number of significant factors for the sediment of Dalian Bay.

PAHs 9 (Polycyclic Aromatic Hydrocarbons)	Coefficient of Determination					
	1	2	3	4	5	6
acenaphthylene (AcNP)	0.50	0.65	0.99	1.00	1.00	1.00
acenaphthene (AcN)	0.49	0.98	0.98	0.99	1.00	1.00
fluorene (Fl)	0.81	0.92	1.00	1.00	1.00	1.00
phenanthrene (PhA)	0.77	0.89	0.91	0.91	0.99	1.00
anthracene (An)	0.81	0.83	0.84	0.93	0.98	1.00
fluoranthene (FlA)	0.88	0.88	0.93	0.94	0.99	0.99
pyrene (Py)	0.92	0.97	0.97	0.98	0.99	0.99
benzo[a]anthracene (BaA)	0.92	0.96	0.96	0.97	0.98	1.00
chrysene (Chr)	0.90	0.99	0.99	0.99	0.99	0.99
benzo[b] + [k]fluoranthene (Bb + kF)	0.89	0.92	0.99	0.99	0.99	1.00
benzo[e]pyrene (BeP)	0.88	0.94	0.96	0.96	0.98	0.99
benzo[a]pyrene (BaP)	0.90	0.94	0.97	0.97	0.97	0.99
indeno[1,2,3-cd]pyrene (IP)	0.76	0.80	0.93	0.98	0.98	1.00
dibenzo[a,h]anthracene (DahA)	0.79	0.88	0.93	0.98	0.98	0.99
benzo[g,h,i]perylene (BghiP)	0.66	0.70	0.86	0.94	0.94	1.00
Cumulative variance (%)	90.70	95.82	98.40	99.03	99.56	99.87
Exner function	0.0820	0.0470	0.0292	0.0236	0.0085	0.0039

**Table 2:** Cosine similarity of modeled and literature source profiles [4,19].

Factor Loadings	Literature PAH Profiles					
	Power Plant	Residential Coal	Coke Oven	Gasoline Engine	Diesel Engine	Traffic Tunnel
1 of 2	0.87	0.72	0.89	0.92	0.82	**0.94**
2 of 2	0.80	**0.96**	0.70	0.65	**0.92**	0.81
1 of 3	0.86	0.71	0.90	0.92	0.80	**0.94**
2 of 3	0.82	0.83	0.69	0.72	**0.85**	0.78
3 of 3	0.73	**0.93**	0.57	0.52	0.86	0.68

Bold face type indicates probable PAH source profile as discussed in the text.
